# Online vs. Classroom Learning: Examining Motivational and Self-Regulated Learning Strategies Among Vocational Education and Training Students

**DOI:** 10.3389/fpsyg.2019.02795

**Published:** 2019-12-19

**Authors:** Carla Quesada-Pallarès, Angelina Sánchez-Martí, Anna Ciraso-Calí, Pilar Pineda-Herrero

**Affiliations:** ^1^Serra Húnter Fellow, Department of Applied Pedagogy, Universitat Autònoma de Barcelona, Bellaterra, Spain; ^2^Department of Systematic and Social Pedagogy, Universitat Autònoma de Barcelona, Bellaterra, Spain; ^3^Department of Methods of Research and Diagnosis in Education, Universitat de Barcelona, Barcelona, Spain

**Keywords:** Vocational Education and Training, motivation, self-regulated learning strategies, online learning, learning mode

## Abstract

Numerous studies have been conducted to explore students’ employment of motivational and self-regulated learning strategies (SRL). Research highlights the importance of having motivated students equipped with strategies that help them self-regulate their learning, this being highly important when learning is acquired through online learning programs. Nonetheless, such research has been scarce with Vocational Education and Training (VET) students; this is the gap in the literature this paper aims to address. The article analyzes the degree to which VET students employ motivational and SRL strategies by comparing them according to the learning mode chosen. To achieve this, a quantitative approach was adopted to carry out a cross-sectional study. A total of 577 first-year VET students responded to an online questionnaire based on some of the motivational and SRL strategies scale included in Pintrich’s model. Statistical analyses were applied to test two hypotheses. Pintrich’s model was validated through a confirmatory factor analysis considering its application to Catalan VET students for the first time. The results reveal significant differences between classroom and online students in terms of levels of metacognitive self-regulation and effort regulation when starting a VET program. However, this difference might not be entirely explained by the learning mode chosen. The findings of this study will provide VET researchers and practitioners with a greater understanding of their students’ characteristics when starting the program and the means to develop strategies that ensure their engagement throughout the course.

## Introduction

There has been an international explosion of online learning and products in recent years, including the online delivery of Vocational Education and Training (VET) programs (Brennan et al., 2001). Online VET changes the relationship between educators and learners, interaction with the learning content, and among learners themselves, and contributes to alleviating time and space barriers. Students enrolled on an online VET course have the freedom to acquire learning whenever and wherever they have the opportunity to. This situation allows students to control how they learn, the pace of their learning, and how to balance their daily tasks with the need to attend the course (Graham, 2006; Alkis and Taskaya, 2018). However, it is important to remember that in order “to succeed in autonomous online learning environments, it helps to be a highly motivated, self-regulated learner” (Artino and Stephens, 2009; Gegenfurtner et al., 2019).

This paper analyzes the degree to which students employ motivational and SRL strategies a few months after beginning a VET program. The aims of the study implemented to ascertain this were to provide VET researchers and practitioners with a greater understanding of their students’ characteristics when starting the program and the means to develop strategies that ensure their engagement throughout the course.

Motivational and self-regulated learning (SRL) strategies employed by VET students have been underexplored in research, making it difficult to have a clear idea of what these might comprise (Savoie-Zajc et al., 2010). Various researchers (Cardinal, 2010; Cournoyer et al., 2015; Dubeau et al., 2017) have suggested that the low number of studies in this area could be related to the reputation that VET programs have of attracting less motivated students. Therefore, one reason for conducting this research is that to the best of our knowledge no studies have previously analyzed differences in students’ motivational and SRL strategies with regard to the different types of VET learning available (traditional or online).

Much research has been conducted in education studies with regard to the role academic motivation plays in student success (e.g., Brackney and Karabenick, 1995; Credé and Phillips, 2011). Results show that the two variables are highly correlated (Müller, 2008; Park and Choi, 2009; Wang et al., 2013; Alkis and Taskaya, 2018). Indeed, Chen and Jang (2010) found high attrition rates to be negatively correlated with motivation in online learning environments. This can be linked to Rovai and Jordan’s (2004) findings that online learning is not suitable for all students and that students succeeding in an online learning environment must be both highly motivated and able to regulate their own learning (Garrison, 2003; Artino and Stephens, 2009).

In the field of education, various motivational theories are employed in relation to academic motivation (Deci and Ryan, 1985; Berndt and Miller, 1990; Pintrich, 1991; Zimmerman et al., 1992; Meece and Holt, 1993), even if these do include common variables such as intrinsic motivation, extrinsic motivation, and self-efficacy, among others. Clark (1998) suggested that in order to obtain high commitment in a task, *task value* is the most important of the motivational factors, whereas Aristeidou et al. (2017) and Jung and Lee (2018) found task value to be associated with learning engagement and therefore learners’ learning performance in online learning (Zhang and Liu, 2019). The concept of task value derives from expectancy-value theory (Eccles and Wigfield, 1995, 2002), understood as the extent to which tasks meet individual needs in pursuing a goal. In other words, how far students perceive the task they are doing is important and useful for their future plans or goals.

Studies on the concept of task value have obtained various results: if the task is of little value to students, they will not engage in it (Eccles and Wigfield, 1995; Neuville, 2004); high perceptions of task value positively correlate with course grades (Brackney and Karabenick, 1995; Linnenbrink and Pintrich, 2003; Liem et al., 2008; Najafi et al., 2018); and task value is a significant predictor of course completion (Pachlhofer and Vander Putten, 2014; Cho and Heron, 2015; Vanslambrouck et al., 2018). In a longitudinal study, Lee (2015) found that task value in online learning remained stable during an entire semester, showing that if students are helped to recognize the value of a task at the beginning of a course, they will stay engaged for the duration of it. However, in a study comparing blended learning with online learning, Alkis and Taskaya (2018) only found task value to be a significant predictor of academic success in the former.

In the context of VET, Radovan and Radovan (2015) found that task value increased among students when they were provided with realistic job-related tasks that put the learning they had acquired into practice. Therefore, when provided with powerful and meaningful learning environments, students perceive their learning tasks as being more valuable. Another study carried out with VET students by Dubeau et al. (2017) identified four patterns related to motivational and individual characteristics (exceptional, talented, low-achieving and drop-out), perception of task value being significantly higher among *exceptional students*.

Pintrich and DeGroot (1990) found not only motivational variables but also cognitive processes such as learning strategies to be important in explaining students’ academic success, as stated by Martínez and Galán (2000). Specifically, in online environments, high levels of academic motivation and self-regulation were found to be due to the autonomous nature of online learning compared to traditional classroom contexts (Artino and Stephens, 2009). Despite its theoretical fuzziness, self-regulation is acknowledged as a multidimensional and process-oriented research construct coming from educational psychology (Kaplan, 2008; Prinz, 2019). Zimmerman (2000) regarded self-regulation as thoughts, feelings, and actions that are planned and modified to the fulfillment of personal goals. Hence, there is “no one set of cognitive, metacognitive, motivational, and behavioral strategies that constitutes the desirable mode of engagement in every setting and task” (Kaplan, 2008, 483).

Thus, the first hypothesis of this study is as follows.

H1. VET students employ motivational and SRL learning strategies in differing degrees according to the learning mode chosen (classroom or online). H1a for task value; H1b for metacognitive self-regulation strategies; and H1c for effort regulation strategies.

To be considered “self-regulated,” students must be committed and efficiently control their own learning process (Zimmerman, 2015). However, learners’ self-regulated learning is neither easy nor automatic (Pintrich, 1999). This translates into at least three important qualities: (1) self-observation (monitoring one’s actions and thinking processes); (2) self-judgment (evaluating one’s performance); and (3) self-reactions (one’s response to performance outcomes) (Broadbent and Fuller-Tyszkiewicz, 2018). Other scholars add that holding positive motivational beliefs (positive attributions) regarding one’s capabilities is also required for higher levels of self-efficacy (Boekaerts et al., 2000). In respect of this, SRL is a constructive process that develops with opportunities for self-directed practice over time. It is based on past experiences and personal, behavioral, and environmental factors (Pintrich, 2000; Zimmerman, 2015).

The phenomenon of self-regulation is complex and has been theorized in different ways. Most theories agree in highlighting behavioral, motivational, and cognitive processes as constitutive parts of SRL. First, behavior self-regulation includes students’ control of resources, such as effort regulation, help seeking, and time/study management (Pintrich, 2004). Second, self-regulation of motivation and affect entails controlling and revising motivational beliefs, such as self-efficacy and goal orientation, to meet the demands of a task. And finally, self-regulation of cognition encompasses the control of deep processing strategies that lead to better learning and performance.

In addition, several models of SRL (Sitzmann and Ely, 2011) have recently been analyzed and compared by Panadero (2017). All models agree that SRL is cyclical and composed of different phases and subprocesses. However, the labels and processes in each phase differ from one model to another. Of such models, Pintrich’s (2000). SRL model (2000) had a highly significant impact in the field and is widely known for its development of an instrument to measure SRL: the Motivated Strategies for Learning Questionnaire. The model classifies phases that other SRL models commonly share and areas for SRL (Lee et al., 2019), dividing SRL into the following four phases: (1) Forethought, planning and activation of prior knowledge of the task, the context, and the self in connection with the task; (2) Monitoring processes; (3) Control and regulation of different parts of the task, the context, and the self; and (4) Reaction and reflection on the task, the context, and the self – each also with four different areas for regulation: cognition, motivation and affect, behavior and context. The degree of student learning varies according to key self-regulatory processes. Pintrich (2004) stated that these SRL strategies were systematically directed toward the achievement of learning goals and divided them into three groups: (1) cognitive, (2) metacognitive, and (3) resource management. Cognitive strategies such as selective attention, decoding or structuring enable students to fuse new and existing information (Richardson et al., 2012). Metacognitive strategies refer to an awareness of learning procedures so as to be able to establish goals; thus, they are related to mentally representing learning goals, designing action plans, self-monitoring progress and evaluating goal achievement. Finally, resource management strategies require students to use social and their own resources to persist when confronted with a task (Richardson et al., 2012). Examples of students’ regulatory strategies for controlling resources other than their cognition include managing their time, effort and study environment, as well as the use of peer, teacher, and other help-seeking learning strategies such as benefiting from a study group.

Within resource management strategies and SRL behavioral capacities (Pintrich, 2004), regulatory processes focus on how students best implement effort toward the accomplishment of academic tasks (Zeidner and Stoeger, 2019). In this sense, effort regulation occupies a key role in SRL and is understood as a learning strategy that entails self-managing motivation or persistence (Theus and Muldner, 2019). It is related to conscientiousness and academic self-efficacy.

Self-regulation also involves the transfer of self-regulation processes (knowledge, skills, and attitudes) to different learning situations and contexts, including work and leisure (Boekaerts, 1999, cited in Liveris and Cavanagh, 2012). In fact, through cyclical phases that explain the interrelation of the metacognitive and motivational processes involved in SRL at the individual level, students acquire what is known as self-regulatory competency (Zimmerman, 2000).

Due to the proliferation of digital environments, students now have more opportunities for interaction and practice, however, the design of digital learning contexts needs careful attention to ensure that the self-learning process is optimized (Ting and Chao, 2013). Likewise, there is a lack of evaluations measuring the impact of SRL on students in digital environments (Pérez-Álvarez et al., 2018). Moreover, the degree to which learners use SRL strategies may mediate the effects of dispositional characteristics and psychosocial contextual influences on academic performance in highly autonomous instructional settings. This has been understudied, however, and warrants further empirical investigation because it could have important educational implications for instructors (Artino and Stephens, 2009).

In summary, various studies have pointed out that online students need to employ motivational and SRL strategies more extensively in order to succeed academically. This raises a question regarding what variables -learning mode among them- explain differing degrees of motivational and SRL strategies employed by VET students when starting a program. Thus, the second hypothesis of our study is as follows.

H2. The learning mode chosen by VET students is a key variable when explaining the degree to which they employ motivational and SRL strategies when starting the program.

As mentioned, the general aim of this paper is to analyze the degree to which VET students employ motivational and SRL strategies a few months after beginning the program. In order to achieve this, the aim was divided into two specific goals: (1) to validate the adaptation of three scales measuring task value, effort regulation and metacognitive self-regulation; and (2) to identify differences in students’ motivational and SRL strategies depending on the learning mode they have enrolled for (classroom or online). The results are presented in line with the aims outlined above.

## Materials and Methods

### Design and Procedure

In order to respond to the research aims, a cross-sectional design was used. An on-line questionnaire was administered to a sample of classroom and online VET students during their first academic year, in two different ways.

For classroom VET students, the course coordinators or group tutors–henceforth tutors–were in charge of administration, which took place in the classroom, in a group setting, on a day and at a time agreed with the research team. Tutors provided their students with the link to the online questionnaire in class or *via* Moodle; students could either access the tool using their mobile phone or administration took place in the IT classroom if they were not allowed to bring their phones to school. Tutors were also responsible for reminding students that the questionnaire was completely anonymous and checking the last screen of the questionnaire to ensure the tool had been answered in full.

For online VET students, the school directors uploaded the links to the online questionnaire to the virtual learning environment–Moodle–and gave the students 2 weeks to respond to it. After a week, they sent a gentle reminder to all students in order to obtain more responses.

In both cases (classroom VET and online VET students) the scales were applied in the same order and through the same online platform. Response time varied largely, depending on question routes in the part regarding demographic information; but typically, it took 15 min to answer the entire questionnaire.

The Research Ethics Committee CER (FCES-2018-04) belonging to the International University of Catalonia (UIC Barcelona) approved the research design and implementation, including all consent procedures followed in the study. All participants were at least 16 years old and informed that they could refuse participation in the research or withdraw at any moment. The questionnaire was anonymous and participants’ informed consent was implied through survey completion.

### Participants

A purposive sampling technique was used to select potential participants. First, 10 VET programs were selected according to different criteria: the research team sought to include programs from both of the levels offered within the initial Spanish VET system (ISCED 3B and 5); all included programs had to be offered in both classroom and online modes and had to match the priority economic sectors, which were identified by experts in a previous phase of the research. It is important to clarify that although students were completely free to decide which learning mode they wanted to follow, this decision depended on many factors, such as the availability of the program in online mode, working while studying or family responsibilities.

Once the VET programs had been selected, we proceeded to select 39 public schools that ran these programs: these comprised four schools per program–one in each of the four Catalan provinces–except for one of the programs, which was only offered in two schools (leaving a total of 38 schools) plus one online school offering all the selected VET programs. Private schools were excluded from the sample because of the diversity of their VET teaching models, especially the online version.

The final sample was composed of 577 first-year VET students, out of a population of 92,125 pupils enrolled on VET programs in public schools in Catalonia (8,764 online students, 83,361 classroom VET students for the 2017–2018 school year), according to the latest public data available from the Catalan Education Department (Departament D’educació [DE], 2018). Table 1 shows the main characteristics of the sample (valid cases).

**TABLE 1:** Description of the sample.

**Variable**	**Distribution**
Gender	42.5% females; 56.2% males; 1.4% other or do not want to answer.
Age	Mean 24.89 years (20.65 years for classroom VET; 37.96 for online VET); standard deviation 9.756 years (4.71 for classroom VET; 9.67 for online VET).
Type of program	75.4% classroom VET; 24.6% online VET.
Program	56.3% technology sector; 43.2% health and care sector; 0.5% other sectors.
ISCED level	42.3% level 3B; 57.2% level 5.
Prior work experience	29.8% no; 70.2% yes (among whom, 39.3% had work experience related to the VET program they were attending).
Main reason to enroll on VET program	28.8% personal interest; 25% to find a job in this sector; 24.3% to progress in my professional career; 9.7% to get a certificate; 7.5% other reasons; 4.9% to demonstrate to myself that I have the ability.

### Tools

The research tool comprised an *ad hoc* questionnaire in Catalan based on various validated scales. The decision to translate the questionnaire into Catalan was based on the language policy of Catalonia: as an autonomous region of Spain, Catalonia has some educational provisions particular to its region, including using Catalan as the language of instruction.

The questionnaire included a first part with questions asking for demographic information, mainly multiple-choice items (age, gender, school pathways, work experience), and a question about the main reason for enrolling on the VET program. The latter was a multiple-choice item with responses adapted from the Spanish version of the Vermunt Inventory of Learning Styles (Martínez-Fernández and Vermunt, 2013), specifically from the following learning orientations: personally oriented, certificate-oriented, self-test oriented, and vocation-oriented.

The second part of the instrument was composed of questions on cross-disciplinary skills (critical thinking, teamwork and communication), as well as the three variables related to motivational and SRL strategies: task value, effort regulation and metacognitive self-regulation. Lastly, students had to evaluate their accomplishment of five key technical/professional skills specifically related to their VET program.

All three scales related to motivational and SRL strategies -task value, effort regulation and metacognitive self-regulation- were measured using our own adaptation of the corresponding factors taken from the Motivated Strategies for Learning Questionnaire (MSLQ) (Pintrich, 1991). A selection of only some of the factors was required, since the questionnaire is very long and complex; furthermore, the MSLQ is modular, and it is very common for researchers to only use parts of it rather than the entire instrument (Holland et al., 2018). In order to choose the most relevant scales, we considered metacognitive self-regulation and effort regulation to be the strategies most related to the concept of “learning to learn,” this emerging as one of the key competences for apprentices in a previous phase of the research based on interviews with stakeholders. In addition, task value was also included as it is one of the key motivational factors that can impact learning engagement (Aristeidou et al., 2017; Jung and Lee, 2018; Zhang and Liu, 2019), and therefore course completion (Pachlhofer and Vander Putten, 2014; Cho and Heron, 2015; Vanslambrouck et al., 2018). Since online courses generally have higher failed retention rates than classroom settings (Herbert, 2006; Smith, 2010; Bawa, 2016), the examination of this variable could be especially relevant for online VET students, who would probably need to employ a high degree of task value in order to successfully complete their studies.

To ensure the transferability of results, a backward translation procedure was followed. Two native Catalan speakers who are fluent in English translated the items into Catalan from the original English version and also adapted them to fit both the VET and online education contexts. A third native Catalan speaker checked and standardized both translations; when doubts arose, the Spanish version (Martínez and Galán, 2000) was consulted for the available items. In order to make the whole tool more uniform, the original response scale (a 7-point Likert scale from “Not at all true of me” to “Very true of me”) was converted into a five-point frequency scale (from “Never” to “Always”). Table 2 presents the three variables, with the number of items for each variable and an example item. Cronbach’s Alphas are also reported for the original Pintrich (1991) scales.

**TABLE 2:** Analyzed variables, with number of items, example items and alpha coefficient.

**Scale**	**Dimension**	**Variable**	**No items**	**Example item**	**Cronbach’s Alpha**	**Mean (Standard Error)**
Motivation scale	Value components	Task value	6	I think the course material in this class is useful for me to learn.	0.90	4.17 (0.78)
Learning strategies scale	Resource management strategies	Effort regulation	4	I often feel so lazy or bored when I study for this class that I quit before I finish what I planned to do (reversed).	0.69	3.71 (0.82)
	Cognitive and metacognitive strategies	Metacognitive self-regulation	12	I ask myself questions to make sure I understand the material I have been studying in this class.	0.79	3.33 (0.75)

According to Pintrich’s (1991) model, task value is defined as students’ evaluation of how interesting, how important, and how useful the task and the course material are. Effort regulation reflects a commitment to completing one’s study goals, even when students encounter difficulties or distractions. Metacognitive self-regulation refers to exercising control over cognition and learning; it includes three general processes: planning, monitoring, and regulating.

### Data Analysis

Since the original Pintrich (1991) scales had already been translated into Catalan and adapted to fit the VET context, their validation was required. In order to obtain evidence of validity based on the internal structure, a confirmatory factor analysis (CFA) was performed using the AMOS v.23 software. The original structure (Pintrich and DeGroot, 1990) was tested, with three correlated factors. After data depuration and inverting reversed items, a maximum likelihood estimation method was used (with regression imputation for missing values). Some readjustments were made to the final model by observing the regression weights and modification indexes.

Normality assumption of the factor scores was checked using the Kolmogorov-Smirnov test of normality, skewness and kurtosis intervals, visual inspection of normal and detrended Q-Q plots. The Kolmogorov-Smirnov tests showed none of the three variables to be normally distributed (*p* < 0.0001 for task value and effort regulation; *p* = 0.011 for metacognitive self-regulation). Task value and effort regulation distributions revealed negative asymmetry (skewness statistics −1.688 and −0.307, with standard error of 0.104); moreover, task value showed a leptokurtic distribution (kurtosis statistic 3.440, with standard error of 0.207). Also, the violation of normality for metacognitive self-regulation was due to multiple peaks and dips. Table 2 presents the mean and standard error values for each of these variables.

In order to test H1, after checking the violation of normality assumption, Mann-Whitney U tests were performed. Finally, to test H2, three multiple regression models were performed, using the three motivational and SRL strategies as dependent variables; student profile and program type (classroom or online VET) were included. The stepwise method was used for each model. Independent variables (except for age) were encoded as dummy variables (0, 1). Following Lévy and Varela’s (2003) recommendation, the multiple regression model analysis applied in this study did not aim to establish prediction, but to facilitate our understanding of which variables - learning mode among them- explain the different degrees of motivational and SRL strategy employed by VET students upon commencing the course.

## Results

### Validation of Motivational and SRL Strategies Scales

After testing the first model, simulating Pintrich’s (1990) original, the results suggested that the latent factor of metacognitive self-regulation had small regression weights on Items 1 and 8 (for Item 1, γ = 0.159 with *p* < 0.0001, and for Item 8, γ = 0.066 with *p* = 0.151). After analyzing item content and wording, we noticed that the two questions had a similar structure: both were reversed and had the word “often” in them. We therefore decided to eliminate these items from the analysis, since they did not have the same effect as the originals, possibly due to the translation into Catalan, which led to changes in the structure of the sentences.

**FIGURE 1:**
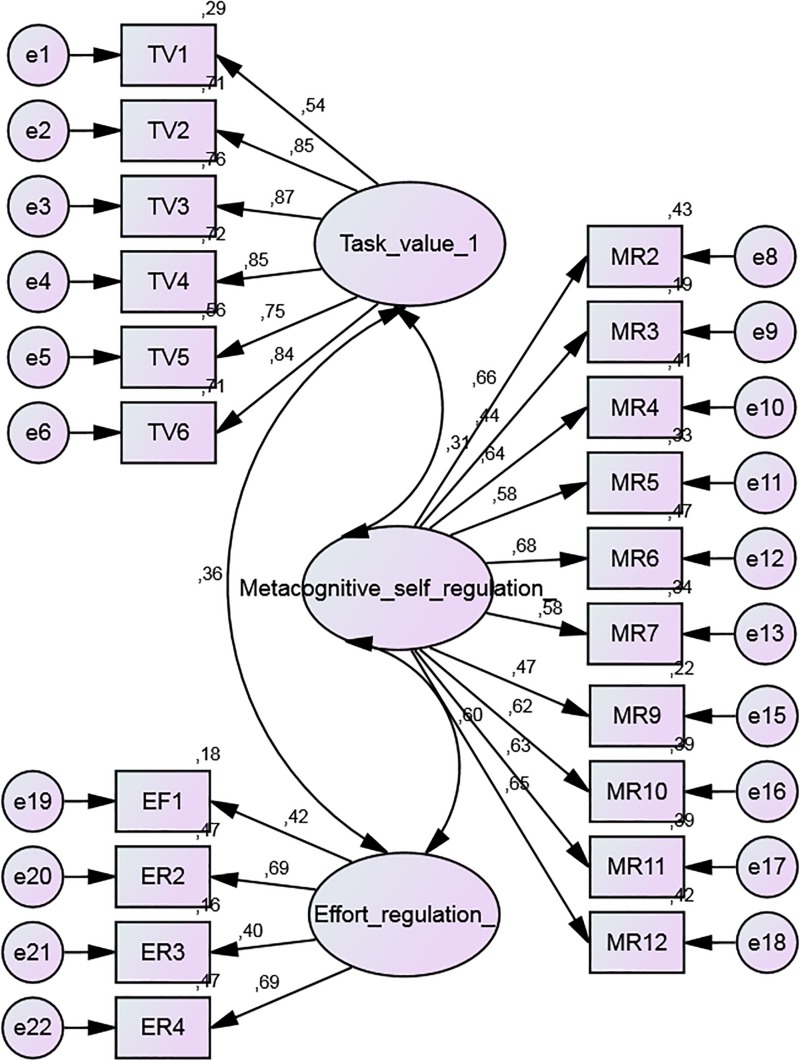
Final CFA model (standardized estimates).

Following this readjustment, the final model improved the χ^2^ (from χ^2^ = 921,387 to χ^2^ = 573.409). Other fit indices appeared to be marginally acceptable (CMIN/DIF = 3.434; CFI = 0.910; TLI = 0.898; RMSEA = 0.065). Figure 1 presents the standardized estimates and correlations among factors, and Table 3 shows the non-standardized regression weights.

**TABLE 3:** Final CFA model (non-standardized regression weights).

**Item**	**Latent factor**	**Estimate**	**S.E.**	**C.R.**
TV6	Task value	0.964^∗∗∗^	0.037	26.198
TV5	Task value	0.856^∗∗∗^	0.040	21.483
TV4	Task value	0.960^∗∗∗^	0.036	26.597
TV3	Task value	1.000		
TV2	Task value	0.910^∗∗∗^	0.034	26.403
TV1	Task value	0.627^∗∗∗^	0.046	13.740
MR2	Metacognitive self-regulation	0.984^∗∗∗^	0.082	11.984
MR3	Metacognitive self-regulation	0.610^∗∗∗^	0.069	8.876
MR4	Metacognitive self-regulation	0.978^∗∗∗^	0.083	11.782
MR5	Metacognitive self-regulation	1.000		
MR6	Metacognitive self-regulation	1.086^∗∗∗^	0.088	12.315
MR7	Metacognitive self-regulation	0.870^∗∗∗^	0.079	11.047
MR9	Metacognitive self-regulation	0.643^∗∗∗^	0.069	9.347
MR10	Metacognitive self-regulation	0.844^∗∗∗^	0.073	11.565
MR11	Metacognitive self-regulation	1.019^∗∗∗^	0.088	11.612
MR12	Metacognitive self-regulation	0.988^∗∗∗^	0.083	11.917
ER4	Effort regulation	1.591^∗∗∗^	0.205	7.763
ER3	Effort regulation	1.000		
ER2	Effort regulation	1.672^∗∗∗^	0.216	7.756
ER1	Effort regulation	1.154^∗∗∗^	0.180	6.421

*^∗∗∗^*p* < 0.0001.*

The analysis of the standardized residual covariances revealed some local misfits (by detecting some values |1.96|), particularly affecting the latent factor effort regulation. Likewise, the modification indexes suggested freeing the regression weights on effort regulation items from other factor items. These suggestions were not considered since the theoretical model did not support them, however, a revision of these items is highly recommended.

Finally, Cronbach’s Alpha was α = 0.844 for metacognitive self-regulation; α = 0.665 for effort regulation; and α = 0.902 for task value. Again, these results showed the need to improve the effort regulation scale, whereas the other two scales can be considered sufficiently reliable for group-level analysis.

The final instrument is available at https://ddd.uab.cat/record/215267.

### Mean Differences Between Learning Modes Among Vocational Education and Training Students

H1 established that VET students employ different degrees of motivational and SRL learning strategies according to the learning mode chosen (classroom or online)–H1a, H1b, and H1c–. The results are presented below.

Perceptions of task value among online VET students (*Mdn* = 4.33) did not differ significantly from those of classroom VET students (*Mdn* = 4.33) at the beginning of the program, *U* = 28674.50, *z* = -1.287, *p* = 0.198, *r* = -0.054. This means that VET students enrolled on online learning programs have the same perception of task value as those students enrolled on classroom learning programs.

Metacognitive self-regulation levels among online VET students (*Mdn* = 3.50) differed significantly from those of classroom VET students (*Mdn* = 3.30) at the beginning of the program, *U* = 24116.50, *z* = -2.244, *p* = 0.025, *r* = -0.095. This means that at the beginning of a course, VET students enrolled on online learning programs perceive that they employ more highly developed metacognitive self-regulation strategies than those students enrolled on classroom learning programs. The data show a small effect size.

Effort regulation levels among online VET students (*Mdn* = 4.00) differed significantly from those of classroom VET students (*Mdn* = 3.50) at the beginning of the program, *U* = 18745.50, z = -5.623, *p* < 0.001, *r* = -0.239. This means that at the beginning of a course, VET students enrolled on online learning programs perceive that they employ more highly developed effort regulation strategies than those students enrolled on classroom learning programs. The data show a small effect size.

Considering the mean comparison, we refuted H1a and confirmed H1b and H1c.

### Variables That Explain Degrees of Motivational and Self-Regulated Learning Strategies Employed by Vocational Education and Training Students

The study considered the second hypothesis: H2. The learning mode chosen by VET students is a key variable when explaining the degree to which they employ motivational and SRL strategies when starting the program. The statement was based on the idea that if online learners need to employ more motivational and SRL strategies in order to succeed academically, then it is important to determine which variables -learning mode among them- explain the different degree to which VET students employ motivational and SRL strategies at the beginning of the course.

To this end, we executed a multiple regression model using the motivational and SRL strategies as dependent variables. The independent variables were: student profile—age, male student, female student, pathways, having prior work experience, having prior work experience related to the VET program; classroom as learning mode; main reason for enrolling on VET program—personal interest, to find a job in this sector, to progress in my professional career, to get a certificate, to demonstrate to myself that I have the ability; and the other motivational and SRL strategies not used as dependent variables.

The first multiple regression model was executed using task value as the dependent variable. After five steps, the model obtained an adjusted *R*^2^ of 0.138. This means that although metacognitive self-regulation, effort regulation, the motivation to progress in one’s professional career, personal interest in the content of the VET program, and having professional experience (in general) were variables in the resulting model, they only explained 13.8% of perceived task value among VET students when starting a program. Table 4 shows the coefficients of the resulting model, in which the chosen learning mode did not emerge as a significant factor in explaining perceived task value among VET students when starting a program.

**TABLE 4:** Multiple regression model coefficients, using Task value as the dependent variable.

	***B***	***SE B***	**β**
**Step 1**			
Constant	3.16	0.18	
Metacognitive self-regulation	0.31	0.05	0.29^∗∗^
**Step 2**			
Constant	2.82	0.21	
Metacognitive self-regulation	0.24	0.06	0.23^∗∗^
Effort regulation	0.15	0.05	0.16^∗^
**Step 3**			
Constant	2.84	0.21	
Metacognitive self-regulation	0.22	0.06	0.21^∗∗^
Effort regulation	0.15	0.05	0.16^∗^
Reason to enroll: To progress in my professional career	0.21	0.09	0.12^∗^
**Step 4**			
Constant	2.69	0.21	
Metacognitive self-regulation	0.23	0.06	0.22^∗∗^
Effort regulation	0.15	0.05	0.16^∗^
Reason to enroll: To progress in my professional career	0.30	0.09	0.16^∗^
Reason to enroll: Personal interest	0.24	0.09	0.14^∗^
**Step 5**			
Constant	1.49	0.55	
Metacognitive self-regulation	0.23	0.06	0.22^∗∗^
Effort regulation	0.15	0.05	0.16^∗^
Reason to enroll: To progress in my professional career	0.29	0.09	0.16^∗^
Reason to enroll: Personal interest	0.24	0.09	0.14^∗^
I have professional experience (in general)	1.21	0.52	0.11^∗^

*Adjusted *R^2^* = 0.083 for Step 1, Δ*R^2^* = 0.019 for Step 2 (*p* = 0.003), Δ*R^2^* = 0.011 for Step 3 (*p* = 0.015), Δ*R^2^* = 0.014 for Step 4 (*p* = 0.007), Δ*R^2^* = 0.010 for Step 5 (*p* = 0.019), *^∗∗^p* < 0.001 *^∗^p* < 0.05.*

When using metacognitive self-regulation as a dependent variable, four steps were needed to obtain an adjusted *R*^2^ of 0.228. The variables effort regulation, task value, not having a personal interest in the content of the VET program (negative correlation) and being female were key variables that only explained 22.8% of perceived metacognitive self-regulation in VET students. Again, the chosen learning mode was not one of the significant variables in the model. Table 5 presents the coefficients of this model.

**TABLE 5:** Multiple regression model coefficients, using Metacognitive self-regulation as the dependent variable.

	***B***	***SE B***	**β**
**Step 1**			
Constant	1.973	0.161	
Effort regulation	0.366	0.042	0.408^∗∗^
**Step 2**			
Constant	1.341	0.215	
Effort regulation	0.320	0.042	0.357^∗∗^
Task value	0.192	0.044	0.203^∗∗^
**Step 3**			
Constant	1.411	0.214	
Effort regulation	0.304	0.042	0.338^∗∗^
Task value	0.199	0.044	0.211^∗∗^
Reason to enroll: Personal interest	−0.253	0.074	−0.153^∗^
**Step 4**			
Constant	1.444	0.213	
Effort regulation	0.293	0.042	0.326^∗∗^
Task value	0.195	0.044	0.206^∗∗^
Reason to enroll: Personal interest	−0.254	0.074	−0.154^∗^
Female	0.134	0.067	0.090^∗^

**Adjusted R^2^* = 0.164 for Step 1, Δ*R^2^* = 0.037 for Step 2 (*p* < 0.001), Δ*R^2^* = 0.021 for Step 3 (*p* = 0.001), Δ*R^2^* = 0.006 for Step 4 (*p* = 0.047), *^∗∗^p* < 0.001 *^∗^p* < 0.05.*

The last regression model took effort regulation as the dependent variable. The resulting model obtained an adjusted *R*^2^ of 0.246 in only three steps. This model comprised metacognitive self-regulation, student age, and task value. These three independent variables were able to explain 24.6% of the variance in the effort regulation variable, a higher percentage than the other three models. Table 6 offers the coefficients of the model obtained. Again, the chosen learning mode was not a key variable in this model.

**TABLE 6:** Multiple regression model coefficients, using Effort regulation as the dependent variable.

	***B***	***SE B***	**β**
**Step 1**			
Constant	2.252	0.177	
Metacognitive self-regulation	0.454	0.052	0.408^∗∗^
**Step 2**			
Constant	1.837	0.183	
Metacognitive self-regulation	0.402	0.050	0.361^∗∗^
Age (in years)	0.022	0.004	0.275^∗∗^
**Step 3**			
Constant	1.465	0.233	
Metacognitive self-regulation	0.366	0.052	0.329^∗∗^
Age (in years)	0.021	0.004	0.263^∗∗^
Task value	0.124	0.049	0.117^∗^

**Adjusted R^2^* = 0.164 for Step 1, Δ*R^2^* = 0.072 for Step 2 (*p* < 0.001), Δ*R^2^* = 0.011 for Step 3 (*p* < 0.001), *^∗∗^p* < 0.001 *^∗^p* < 0.05.*

The results from all three models refuted the second hypothesis (H2).

## Discussion

The evidence suggests that motivation and SRL strategies are important in determining academic success in any educational stage and process, including in VET studies (Wang et al., 2013; Alkis and Taskaya, 2018). Online learning environments open up new avenues for VET students because they are not seen merely as tools to support learning, but as dynamic settings that are flexible, attractive and interactive, and make lifelong learning possible in any professional field (Brennan et al., 2001). Yet, not all students succeed in online VET. Studies have evidenced that they must be highly motivated and able to regulate their own learning (Garrison, 2003; Artino and Stephens, 2009). Our research aimed to analyze the degree to which VET students employ motivational and SRL strategies, focusing on the two VET modes, online and classroom, in order to understand how students’ characteristics are related to their engagement in the course.

### Validating Task Value, Effort Regulation and Metacognitive Self-Regulation Scales in New Contexts

Regarding the first aim of the research, some evidence of construct validity was obtained for the translated and adapted version of Pintrich’s (1991) scales for analyzing metacognitive self-regulation, task value, and effort regulation among VET students in Catalan.

Our results showed that correlations among factors were quite similar as with the original model constructed by Pintrich (1991). This means that Pintrich’s model is also suitable for analyzing motivational and SRL strategies used by VET students both online and on classroom programs.

Despite the similarities with Pintrich’s (1991) original findings, the results for validity and reliability indicated that a revision of the effort regulation scale is needed. Dunn et al. (2012) faced similar problems when conducting the statistical revaluation of effort regulation and metacognitive self-regulation scales. Thus, more analyses are required to confirm the goodness of fit of SRL scales among VET students in Catalonia, since difficulty and discrimination parameters may have changed. It is crucial that more data are gathered to validate the model in a new context like the current one. In addition, a revision of the translation and adaptation of Items 1 and 8 is required in order to improve them and include them in the model, which would make the results of the Catalan version of the scale more comparable with the original.

Despite the limitations mentioned, this step forward in the validation of scales in itself represents an important achievement that has not been reported previously, because it enables Catalan educational institutions to apply the model to all types of VET studies. This will provide VET instructors with the tools to evaluate the motivational and SRL strategies employed by their students and better adapt their teaching methodology to them.

### Identifying Differences Between Learning Modes

In relation to the second aim of the study, namely, to identify differences in students’ motivational and SRL strategies depending on the learning mode they enrolled on (classroom or online), the results of the Mann-Whitney test were relevant.

Our findings pointed to the fact that VET students enrolled on online learning programs perceived they have more highly developed metacognitive self-regulation and effort regulation strategies than those enrolled on classroom learning programs. This aligns with the results of other studies (Martínez and Galán, 2000; Artino and Stephens, 2009), which showed how important SRL strategies are in explaining students academic success, specifically in online learning (Triquet et al., 2017). Thus, having these strategies when starting a VET program in online mode becomes essential to their success. The last meta-analysis study published by Lee et al. (2019) showed an alignment between of our results and those of several studies on online learners’ success factors and the importance of self-efficacy and SRL strategies. In respect of this, Magen-Nagar and Cohen’s (2017) study proved that learning strategy was a significant mediator for motivation and academic achievement among online high-school students, which constitutes an important contribution to understanding the implications of our results.

Despite what we might have expected, no differences were found between online and classroom VET students when it came to perception of task value. This indicates that online and classroom VET students perceived the value of the task -the course they enrolled on- to be equally important, even though task value has been proven to be one of the most important motivational factors, it being associated with learning engagement and success (Aristeidou et al., 2017; Zhang and Liu, 2019) and a significant predictor of course completion (Cho and Heron, 2015; Vanslambrouck et al., 2018). As Cents-Boonstra et al. (2018) showed, VET students with a higher level of motivation also have a higher level of self-efficacy, which is strongly linked to learning success.

Considering these results, H1a was refuted and H1b and H1c were confirmed. In other words, there were no differences in perception of task value between online and classroom learning VET students, which refuted H1a; but there were differences in perceptions of the metacognitive self-regulation and effort regulation learning strategies employed between these two groups, which confirmed H1b and H1c, online learning VET students perceiving their self-regulated learning strategies to be more developed.

The fact that there were no differences in task value perception between online and classroom VET students reveals an important area for improvement for educational institutions. We expected online students to have a higher perception of task value than classroom students because their learning process is based on a more student-centered and autonomous model and they therefore need more self-motivation. Furthermore, online course satisfaction and its connection with student motivation were also found to be related to academic success in this study (Herbert, 2006), while Lee et al. (2019) review showed that task value and SRL strategies positively affect a sense of academic achievement, motivation and learner behaviors. Online VET instructors and course designers could use this knowledge to improve their programs and thereby foster motivation and success among VET students. Indeed, increasing online VET students’ perception of task value by providing meaningful learning environments could improve students’ motivation and academic success (Radovan and Radovan, 2015; Dubeau et al., 2017).

Approaching these differences by applying multiple regression models that use the factors posited by Pintrich as dependent variables, our findings suggest that more than 75% of the variance of all three models still remains unexplained with the profile variables included. Thus, the regression models obtained do not include all of the variables we need to help us understand which motivational and SRL strategies play an important role when selecting the mode of VET learning. One way to acquire fully explained models could be to use Pintrich’s (1991) complete model; while others could be to measure other profile variables -such as time spent studying course materials, family responsibilities- and cognitive variablessuch as working memory (Torrano and González Torres, 2004), and designing longitudinal studies such as the work done by Lee (2015) using mixed-methods to gain more in-depth knowledge of the reasons behind the results obtained.

It is also interesting to note that the independent variable learning mode classroom or online that students enrolled on for the VET program was not significant in explaining the degree of motivational and SRL strategies employed by these students. Results from the two models allow us to refute our second hypothesis (H2), because we were not able to find any evidence to support the idea that the learning mode chosen by VET students was a key variable when explaining the degree to which they employ motivational and SRL strategies.

This means that even though there were significant differences between these type of students classroom and online in the degree to which they employed metacognitive self-regulation and effort regulation when beginning a VET program, this difference might not be entirely explained by their choice of learning mode. This idea can only be tested by conducting longitudinal studies during their learning period in VET programs similar to the work done previously by Lee (2015) or Throndsen (2011); using various methodological approaches may cover qualitative aspects of their responses that quantitative methodology cannot capture.

### Limitations and Future Directions

Some limitations were identified when considering the implications of this study. The fact that the research was conducted in the Catalan VET context and the tool we applied was in the Catalan language can be seen as a limitation, considering the small size of the Catalan-speaking community. However, given the similarity of the VET system implemented in all Spanish regions—and also in some Latin-American countries—the results could be interesting for other Spanish communities, and the validated tool could easily be adapted to their context. Nevertheless, the specific context of the study generated one important limitation: the difficulty of comparing these results with other studies in the field. Another limitation is related to the non-random study sample, which, despite having an adequate number of participants, meant caution was required when attempting to generalize the results. A further limitation was the validation indices for the scales, which were acceptable but not optimal; more research will be needed to improve the scales.

Due to the little available research on VET students’ motivation and the SRL strategies they employ (Savoie-Zajc et al., 2010), our findings could make an interesting contribution to the field.

Several researchers have suggested (e.g., Cardinal, 2010; Cents-Boonstra et al., 2018; Dubeau et al., 2017; Biemans et al., 2019) that VET programs attract less motivated students, and studies on VET motivational and SRL strategies are therefore not priority areas for exploration. To us, this opens a door to new research, which might focus on the Catalan context using the preliminary validated scales to compare motivation levels and SRL strategies between VET and high-school or university students—these being similar in age but differing in levels of motivation (Martin, 2008).

This article presents only one measurement of motivation and SRL strategies, which could be viewed as a limitation in terms of how far our results contribute to VET research. That said, however, we strongly believe that longitudinal studies are also a missing piece of the puzzle we have started to construct in here. Hence the project extending beyond this paper and including the collection of the same measures over the entire 2-year program; other qualitative techniques will be added to gather as much information as possible to obtain a clearer picture of VET students’ motivation and SRL strategies. Our aim is to determine whether our results are maintained over time, as Lee’s (2015) study did using a longitudinal sample.

In line with this, Lee (2015) has already pointed out that “when measurement on students’ motivation is available in the early stages of a semester, some interventions can be implemented to foster their motivations, thus preventing their dropping out of class” (p.63). Knowing for a fact that this is also the case with Catalan VET students (both in classroom and online learning mode), educators can design and implement advanced tasks that engage students from the very first, since students’ extensive employment of motivational and SRL strategies ensures a high probability of course completion and therefore academic success (Cho and Heron, 2015; Vanslambrouck et al., 2018).

## Data Availability Statement

The datasets generated for this study are available on request to the corresponding author.

## Ethics Statement

The studies involving human participants were reviewed and approved by the Universitat Internacional de Catalunya. Written informed consent from the participants’ legal guardian/next of kin was not required to participate in this study in accordance with the national legislation and the institutional requirements.

## Author Contributions

CQ-P and AC-C contributed conception and design of the study and performed the statistical analysis. AC-C organized the database. CQ-P and AS-M wrote the first draft of the manuscript. CQ-P, AS-M, AC-C, and PP-H wrote sections of the manuscript. All authors wrote sections of the manuscript, contributed to the manuscript revision, read, and approved the submitted version.

## Conflict of Interest

The authors declare that the research was conducted in the absence of any commercial or financial relationships that could be construed as a potential conflict of interest.
